# Exploring the role of mindful eating and self-compassion on eating behaviours and orthorexia in people following a vegan diet

**DOI:** 10.1007/s40519-022-01407-5

**Published:** 2022-05-12

**Authors:** Eliza Kalika, Helen Egan, Michail Mantzios

**Affiliations:** Department of Psychology, Birmingham City University, Curzon Building, Office C325, Birmingham, B4 7DE UK

**Keywords:** Orthorexia Nervosa, Plant-based diet, Mindfulness, Mindful eating, Self-compassion, Vegan diet

## Abstract

Orthorexia nervosa (ON) is a new concept that is more prevalent in vegan populations. ON is characterised by obsessive focus on healthy eating, following restrictive dietary practices and dietary restrictions escalating over time. The aim of this study was to explore problematic eating behaviours in a vegan population, and to explore whether mindful eating and self-compassion have an impact on ON. Two hundred and eighty-seven females and twenty-eight males who followed a vegan diet completed scales in Orthorexia, Self-Compassion, Mindful, Emotional, External and Restraint Eating. The results indicated that individuals with high levels of ON display low levels of self-compassion, and high levels of restrained eating. Moreover, the findings indicated that self-compassion, but not mindful eating, partially mediated the relationship between restrained eating and orthorexia nervosa. The present results contribute to a better understanding of orthorexic eating behaviours in a vegan population, and identifies the mediating capacity of self-compassion. Further implications and future directions are discussed.

**Level of evidence**: Level V, cross-sectional descriptive study.

## Introduction

Recent years have seen a significant increase in individuals who consume mainly plant-based foods, such as vegetarian and vegan diets [1]. Individuals follow a plant-based diet for a number of reasons generally summarised under health, morals, environment and animal welfare [2]. Plant-based diets are often associated with better health outcomes such as lowering risks of cancers, lower body weight and decreased likelihood of cardiovascular disease [3, 4]. Studies have also shown that plant-based (compared to omnivore) diets are more effective for weight loss [5]. Kahleova et al. [6] showed that individuals who are overweight benefited more from a plant-based diet showing improvements in their body weight, fat mass and insulin resistance markers. Not consuming any meat, dairy, or any products that originate from animals benefit individuals on plant-based diets [7, 8], especially since meat availability is highly correlated to prevalence of obesity [9]. Contrary, some research has suggested that individuals who consume a meat-free diets are more likely to develop an eating disorder (e.g., [3]). Evidence indicated that 50% of patients with anorexia nervosa consumed a vegetarian and vegan diet [10] compared to six percent in the general population [11]. The mixed findings in a rapidly developing population mandates further research, as well as potential solutions to problems that may be associated to adopting a plant-based diet.

A rather less explored eating behaviour that is prevalent in plant-based dieting and is bordering to the disordered spectrum of food consumption is orthorexia nervosa (ON) (e.g., [12, 13]). ON is a relatively new concept, and is characterised by a fixation with healthy eating [14]. For a person experiencing ON, manifestations of inflexible eating behaviours ranging from a focus on consuming organic, raw foods to a complete avoidance of any foods that they consider being unhealthy, which might lead to malnutrition [15, 16]. Extreme dieting restrictions necessitate lengthy food preparation and often severely reduce the enjoyment of food [17], creating further psychological implications that are associated to ON. For example, thoughts about only consuming clean and healthy foods and avoiding unhealthy foods may become persistent and intrusive, and distress at breaking food rules may be extreme [17, 18] (Brytek-Matera et al., 2019). There are no diagnostic criteria for ON in the current classification systems [19], and a lack of wider recognition in clinical and medical fields, mandating a greater need for further research as the prevalence for ON varies from 1 to 10% in the general population [20–22].

Research has showed an association between ON and plant-based diets [12, 23, 24], where people who adhere to vegetarian and vegan diets could be at greater risk of developing ON than individuals who consume meat. Following a plant-based diet requires eliminating certain foods and adhering to strict nutritional rules, behaviours that are characteristic of ON [12]. Research has also shown that individuals who follow a vegetarian or vegan diet tend to show more orthorexic behaviours in comparison to meat-eaters [25], however, others suggest that there is no association between plant-based diets and ON [26]. Further research into ON and plant-based diets is needed to establish the nature of this relationship, and while research has mainly focused on vegetarian populations [3, 14, 27], much less is known about the rapidly growing vegan population.

Restrained eating—an eating behaviour observed in plant-based diets [3]—and emotional eating are concepts that are strongly linked to eating disorders [28] and obesity [29, 30]. Restrained eating is characterised by deliberately limiting food intake to control weight [31], and emotional eating is characterised by the consumption of significant amounts of food in response to negative emotions **(**e.g., [32]. Research on restrained eating has indicated that individuals who display signs of ON typically display high levels of restraint [12, 33], while no research to date explored potential associations of ON to emotional eating. Two studies have found that stress, anxiety, and negative emotions were significant predictors of ON in both men and women [34, 35], factors found in other research to predict emotional eating [36, 37], and potentially signposting a relationship between ON and emotional eating. External eating is a concept based on eating in response to external food cues, such as sight, smell, and taste of food regardless of hunger and satiety [38]. There are positive intercorrelations between restrained, emotional, and external eating [29, 38], however, research has mostly focused on emotional and external eating as they appear to co-occur [39]. No research explored the potential link between external eating and ON. Therefore, understanding the role of restrained, emotional and external eating with ON will allow for the development of groundwork that may lead to prevention and intervention programs for individuals who are displaying higher orthorexia symptoms.

These eating behaviours are problematic as they are characterised by consumption of food due to emotional stimuli rather than hunger and satiety [40]. Research has shown that use of mindfulness-based traits and practices improve problematic eating behaviours and eating disorders **(**e.g., [41, 42]). Mindful eating refers to eating in a conscious way, focusing on the present moment with the aim of satisfying hunger [43], however, past definitions of mindful eating have been critiqued as being vague and unreliable. Mantzios [44] proposed that “mindful eating behaviour is defined as the sustained attention on a sensory element of the eating exercise (e.g., the taste), and a non-judgmental (or non-evaluative) awareness of thoughts and feelings that are incongruent to the sensory elements of the present eating experiences.” (p. 3), which is more specific to behaviour itself, rather than decision making that is co-occurring when eating mindfully. He further suggested that “mindful eating behaviour (i.e., sensory experience of eating, non-judgmentally)” is separate “from decision making for mindful eating (i.e., ‘am I still hungry?’ ‘I will not multitask while eating’)” (p.4), which are different elements that may signify different associations and clinical implications for people who are regulating their eating.

Recent research proposed that mindful eating assists in the gradual change of external to internal motivations when eating (such as hunger) when participating in mindful eating interventions [45], promoting heathier eating behaviours [46–49] (Zeros et al. 2021), including an increased intake of fruit and vegetables [50, 51], and reductions in high sugar and energy dense food consumption [52, 53]. Research has also found a negative association between mindful eating and motivations to eat palatable foods [54, 55], fat and sugar consumption [56], and grazing [56]. Pierson et al. [57] found mindful eating to successfully reduce the intensity of food cravings and promotion of control over dietary intake when used as an intervention. Egan and colleagues [58] drew further associations, where mindful eating related negatively to emotional eating (see also [59]), other research highlighted the negative associations to weight gain [60], and the impact of portion size and self-regulation [61], all evidence that could blur the line between health behaviours and the potential promotion of ON when exploring specific dietary populations. To our knowledge, only one study has looked at the effects of mindfulness, rather than mindful eating, on ON; Strahler [62] showed that participants who were in the healthy orthorexia group displayed higher levels of mindfulness (in comparison to an orthorexia nervosa group). Therefore, mindfulness may protect against the development of ON and promote healthier eating, but mindful eating and the attentional and attitudinal aspects directed to the food and the eating experience may propose a different, more relevant outlay to the development of relevant mindfulness-based practices.

Self-compassion—a concept related to mindfulness and mindful eating [56, 63]—is defined as the recognition that suffering, inadequacy and failure are part of a shared human experience [64]. This construct includes three components: self-kindness, common humanity, and mindfulness. In a recent systematic review, Braun et al. [65] provided evidence that self-compassion acts as a protective factor against body dysmorphia and eating pathology. Adams and Leary [66] showed that introducing self-compassion interventions to restrictive eaters reduced their distress-related eating. All of the research has been either focused on general or overweight/obese populations, which makes the findings not generalizable, and difficult to relate to ON. Considering the association of self-compassion to restrictive eating, and the latter to ON, the potential of supporting individuals experiencing higher symptomatology of ON is explored in the present research.

According to Brytek-Matera [14], a number of studies include semi-vegetarians and pescatarians as individuals who consume plant-based diets, which was a barrier to drawing clear conclusions, and therefore, this study focused only on vegans. Furthermore, most of studies on ON focused only on vegetarians, and not vegans, when vegans have more dietary restrictions than vegetarians, signifying the added importance to explore vegans and potential associations to ON. The first aim of this study was to explore problematic eating behaviours, such as emotional eating, restrictive eating, and external eating in a vegan population. The second aim of this study was to explore whether mindful eating and self-compassion have an impact on ON. To our knowledge only one study has looked at effects of mindfulness and ON, but no research has explored the link between ME and self-compassion to ON. In accordance with previous literature, it is hypothesised that emotional and restrained eating will be positively correlated with ON. Moreover, self-compassion and potentially most aspects of mindful eating will be negatively correlated with ON.

## Methods

### Participants

This sample has been consisted of 313 participants (287 females) who were all adults (18 years or over; *M* = 37.44, SD = 12.33) with a mean Body Mass Index (BMI) of *M* = 24.86 kg/m^2^ (SD = 4.87). Participants were recruited through volunteering sampling by advertising the study on several social media platforms, such as Facebook and Instagram. The advertisement on Facebook has been posted in a number of vegan/vegetarian groups requesting individuals to participate in the study. Furthermore, the university’s Research Participation Scheme was also utilised. Participants recruited through the scheme were rewarded with research credits upon completion of the study. To participate in the study, the participants had to be over the age of 18, have good knowledge of English language and not be diagnosed with an eating disorder.

### Materials

*Demographic Information* Participants were asked to report age, gender, ethnicity, weight and height and to select the form of diet that best describes their eating. There were seven options; vegan, lacto-vegetarian, lacto-ove-vegetarian, pescatarian, semi-vegetarian, occasional omnivore and omnivore.

*Düsseldorf Orthorexia Scale (DOS;* [21]) This 10-item self-reported questionnaire that measures the Orthorexic eating behaviour of participant. A 4-point Likert scale from 1 (*this does not apply to me*) to 4 (*this applies to me*) is used. Higher score indicates a more pronounced Orthorexic behaviour. Sample questions include “I have certain nutrition rules that I adhere to” and “I feel upset after eating unhealthy foods”. The current study demonstrates Cronbach’s alpha of 0.83. Furthermore, high retest reliability was shown, where it ranges between 0.67 and 0.79 [21]

*Mindful Eating Behaviour Scale (MEBS;* [67]. The scale contains 17 items that ask about mindful eating in general as well as attention and awareness of mindful eating. This scale utilised a 5-point Likert scale which ranged from 1 (*never)* to 5 (*very often)*, the higher the score the more mindful individual. Sample questions include “I trust my body to tell me when to eat” and “I notice flavours and textures when I’m eating my food”. The MEBS scale demonstrated Cronbach’s alpha of 0.85 for Focused Eating domain, 0.89 for Eating in Response to Hunger and Satiety cues, 0.86 for Eating with Awareness and 0.77 for Eating without Distraction.

*Sussex-Oxford Compassion Scales (SOCS;* [68]) This scale measures compassion for the self and others. For this study, only the compassion for self was measured. This consists of 20 items and responses range from 1 (*not true at all*) to 5 (*always true*), the higher the score the more self-compassion individual has. Sample items include “I connect with my own suffering without judging myself” and “When I’m upset, I can let the emotions be there without feeling overwhelmed”. The present study produced Cronbach’s alpha of 0.82 for Recognising Suffering, 0.83 for Understanding Universality of Suffering, 0.83 for Feeling for the Person Suffering, 0.79 for Tolerating Uncomfortable Feelings and 0.83 for Acting to Alleviate Suffering.

*Salzburg Emotional Eating Scale* (*SEES;* [69]) This scale was developed to measure the changed in food intake in response to emotional experiences. This scale has 20 items which are scored from 1 (*I eat much less than usual*) to 5 (*I eat much more than usual*). SEES assesses four emotional states of happiness, sadness, anger and anxiety. The higher scores indicate that individual eats more when experiencing those emotions. Sample items include “When I feel happy…” and “When I am jealous”. The Cronbach’s alpha for the present study was 0.86.

*Dutch Eating Behaviour Questionnaire (DEBQ;* [38]) DEBQ consists of 33 items, where it measures the eating behaviour of adults and it assesses three dimensions of eating behaviour, such as Emotional Eating, External Eating, and Restrained Eating. It utilises a 5-point Likert scale 1 (*never)* to 5 (*very often)*. Sample items include “If you have put on weight, do you eat less than you usually do” and “If you see others eating, do you also have the desire to eat”. The Cronbach’s alpha for the present study was 0.83 for Diffuse Emotions, 0.89 for Clearly Labelled Emotions, 0.91 for Emotional Eating, 0.84 for External Eating and 0.92 for Restrained Eating.

### Procedure

Ethical approval was obtained from the ethical committee of an institution based in the midland region of the United Kingdom. The study followed ethical practices fully adhering to the British Psychological Society. Participants were recruited via forums and social media groups and were asked to share the study with their contacts. They were presented with information about the study, such as inclusion and exclusion criteria, and the hyperlink to Qualtrics that directed them to the questionnaire. Furthermore, the university Research Participation Scheme was utilised, where individuals gained research credits for participation. Participants had the opportunity to read the information about the research in a Participant Information Sheet, which appeared prior to consenting. Participants consented and created a unique code to identify data in case of withdrawal. Participants were then presented with demographic information, DOS, MEBS, SOCS, SEES and DEBQ. Upon completion, the participant was presented with a debrief form explaining the aims of the study and the procedure in case of withdrawal. Participants only attended one online session, which lasted approximately 20 min.

### Data analysis

**Fig. 1:**
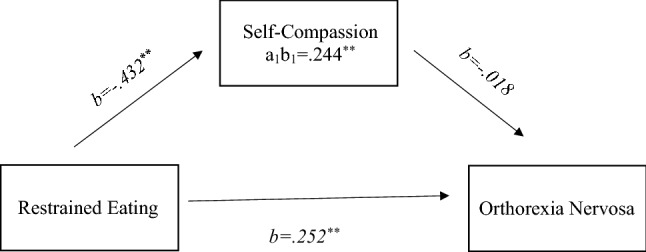
Parallel mediation using standardized regression coefficients to examine the interaction of self-compassion in the relationship between (a) restrained eating and (b) orthorexia nervosa. Notes: *a* is effect of restrained eating on self-compassion; *b* is the effect of self-compassion on orthorexia nervosa; *c*’ is effect of restrained eating on orthorexia nervosa

Data analysis was conducted using SPSS software (version 25.0; IBM Corp., 2017). Means, standard deviations, ranges and internal consistency reliability were calculated for all continuous variables. In addition, descriptive statistics were used to summarise the sociodemographic of the sample. The relationships between BMI, Dutch Eating Behaviour Questionnaire, Salzburg Emotional Eating Scale, Mindful Eating Behaviour Questionnaire, Sussex-Oxford Compassion Scale, Dusseldorf Orthorexia Scale and the vegan population were examined using correlational analysis (see Table 1). A further correlation analysis was conducted to examine the relationships between BMI, DOS and subscales of SOCS (see Table 2). Furthermore, mediation analysis was used to evaluate the indirect effects (via self-compassion) of restrained eating on orthorexia nervosa (see Fig. 1). Hayes’ [70] PROCESS macro (v3.3) was installed on SPSS (version 25.0) and was used to conduct mediation analyses (model 4) using 10,000 bootstrapping resamples to generate 95% bias-corrected confidence intervals for the indirect effect [71]. According to specified guidelines using mediation analyses, Fritz and MacKinnon [72] suggested that a sample size of 462 participants would enable research to observe an indirect effect of a medium–small-sized alpha pathway coefficient (i.e., predictor to mediator) and a medium–small-sized beta pathway coefficient (i.e., mediator to criterion) at 80% power using bias-corrected bootstrapping estimating procedures. Participant’s BMI has been calculated using the information provided by the participant, the alpha level exploring BMI is not possible to be calculated as there is one value that cannot account for any internal consistency.

**Table 1:** Bivariate correlations between DOS, BMI, SOGS, SEES, DEBQ and MEBS

	1	2	3	4	5	6	7
(1) DOS							
(2) BMI	−.090						
(3) SOCS	−.153**	−.156**					
(4) SEES	−.050	.200**	−.033				
(5) EE DEBQ	.046	.266**	−.259**	.617**			
(6) ExtE DEBQ	−.007	.142*	−.209**	.266**	.565**		
(7) RE DEBQ	.397**	.132*	−.307**	−.015	.278**	.167**	
(8) MEBS	.007	.328**	−.401**	.385**	.533**	.385**	.297**

*DOS* Dusseldorf Orthorexia Scale, *SOCS* Sussex Oxford Compassion Scale, *SEES* Salzburg Emotional Eating Scale, *DEBQ* Dutch Eating Behaviour Questionnaire, *MEBS* Mindful Eating Behaviour Scale

*Correlation is significant at the .05 level

**Correlation is significant at the .01 level

**Table 2:** Bivariate correlations between DOS, BMI, SOCS subscales (Recognising Suffering, Understanding Suffering, Person Suffering, Uncomfortable Feelings and Alleviate Suffering)

	1	2	3	4	5	6	7
(1) DOS							
(2) BMI	−.090						
(3) SOCS Recognising Suffering	−.149**	−.014					
(4) SOCS Understanding Suffering	−.146**	−.110	.387**				
(5) SOCS Person Suffering	−.117**	−.182**	.449**	.253**			
(6) SOCS Uncomfortable Feelings	−.086	.−147**	.437**	.314**	.831**		
(7) SOCS Alleviate Suffering	−.110	−.145*	.413**	.275**	.841**	.776**	

*DOS* Dusseldorf Orthorexia Scale, *SOGS* Sussex Oxford Compassion Scale

*Correlation is significant at the .05 level

**Correlation is si

## Results

A multiple correlation analysis has been used to identify which scales (BMI, SOCS, SEES, DEBQ and MEBS) relate to Orthorexia (DOS).

Inter-correlations between DOS, BMI, SOCS, SEES, DEBQ and MEBS are presented in Table 1. Findings suggest that there is a significant negative relationship between ON and SOGS (*p* = 0.007). Furthermore, there is also a significant positive relationship between ON and restrained eating in DEBQ scale (*p* < 0.001). All other correlations were non-significant. On the other hand, BMI had all significant correlations. BMI was negatively associated with SOCS (*p* = 0.006), whereas SEES (*p* < 0.001), emotional eating (*p* < 0.001), external eating (*p* = 0.012), restrained eating (*p* = 0.021) and MEBS (*p* < 0.001), all had a positive relationship with BMI.

A further correlation analysis has been conducted between the ON, BMI and subscales of SOCS, the findings are presented in Table 2. The correlations had showed that ON is negatively associated with Recognising suffering subscale (*p* = 0.009), understanding the universality of suffering subscale (*p* = 0.010) and Feeling for the person suffering subscale (*p* = 0.040). The subscale of Tolerating uncomfortable feelings and acting or being motivated to act to alleviate suffering were non-significant. Furthermore, BMI is negatively associated with Person suffering subscale (*p* = 0.001), uncomfortable feelings subscale (*p* = 0.009) and alleviate suffering (*p* = 0.010). All other subscales were non-significant relating to BMI.

The mediational model analyses (see Fig. 1) use orthorexia as the dependent variable, restrained eating as independent variable, and self-compassion as potential mediator. The c indicated a significant relationship between Restrained eating and orthorexia *b* = 0.252, *p* < 0.001, 95%CI [0.186, 0.318]. Pathway a showed that restrained eating predicted self-compassion *b* = -0.432, *p* < 0.001, 95%CI [−0.583, −0.282]; however, for pathway b self-compassion did not predict orthorexia *b* = −0.018, *p* > 0.05, 95%CI [−0.067, 0.032]. When self-compassion was included in the mediation model, it remained significant *b* = 0.244, *p* < 0.001, 95%CI [0.175, 0.313], this therefore suggests that the relationship between restrained eating and orthorexia is partially mediated by self-compassion.

## Discussion

The aims of the present study were to explore problematic eating behaviours in the vegan population in addition to exploring the impact of mindful eating and self-compassion on ON. Previous research has focused on restrained eating and orthorexia, showing that individuals who display high levels of orthorexia engage in high levels of restrictive eating [12, 18, 27, 33]. Our study confirmed that vegans who show high levels of orthorexic behaviours do engage in more restrictive eating consistent to the observation that individuals who consume a vegan diet have a lot of restrictions as they do not consume any dairy, meat, or products from animal origin [73]. The average BMI for participants is this study was within the healthy range and this is consistent with previous evidence suggesting that vegans are likely to be of lower weight [74] and have lower risk of cancers and cardio-vascular disease [75]. Having a healthy BMI does not preclude the existence of higher occurrences of restrained eating behaviours, which may co-exist with other problematic eating behaviours, such as grazing or binge eating contributing to a higher BMI. Engagement in restrained eating may also escalate over time, leading to psychosocial difficulties, such as not engaging in social events, placing more restrictions on food and severe distress if food rules were broken [17, 27, 33]. Restrictive eating in people who consume a vegan diet is problematic if aligned to orthorexic behaviours.

In accordance with previous studies and what we know about orthorexia, it was hypothesised that individuals who are high in orthorexic behaviours will also have low levels of mindful eating. Our study has shown that there was no relationship between orthorexia and mindful eating, which was surprising as there is a clear attentional aspect that is more evident in following a specific diet, and past research indicated that orthorexia nervosa was negatively correlated with mindfulness [62]. Our findings go against all the studies conducted into mindfulness and mindful eating, and associations to healthier eating [45, 50, 76, 77] and protective values against the development of disordered eating [78]. It was also surprising to find that BMI and mindful eating were significantly positively associated to each other, suggesting that individuals with higher BMIs were more prone to eat mindfully (or vice versa). This is an unexpected finding as previous studies showed that mindful eating was effective in interventions to lose weight and to reduce food intake [79], however, this was conducted in overweight/obese populations and the present sample was of an average weight. Then again other researchers have found similar findings with average weight populations [56, 63], proposing that there may be a differential relationship within vegan populations in the methods employed to weight-regulate when compared to omnivores. Essentially the findings are indicative that there are significant differences in the way mindful eating works for people who consume a vegan diet. The present study has only explored vegans as past research indicated confusion between the definitions (e.g., vegetarians, semi-vegetarians and vegans—[12, 14] and studies combined them to one category [14, 23] creating ambiguity that could lead only to indistinct conclusions about vegans. However, future research should explore more comparatively with people who are not following a vegan diet, and sub-categories, by including clear guidelines to a specific diet to enhance the knowledge around levels of restriction and ON, as well as the association to mindfulness-based constructs.

A further explanation vital in understanding the findings could be the motivations for diet choice [80]. For example, if vegans chose to switch to plant-based diet for ethical/sustainable reasons it could explain the findings as they will be conscious of consuming sustainable and organic produce and not be motivated by weight-regulation. However, a study by Barthels et al. [81] showed that orthorexic eating behaviour in vegans was linked to health motivations rather than animal welfare, consequently shifting the focus to achieving optimal health. Essentially external and internal motivations may signal different aspects and levels of adaptability and health, and potential in enhancing the effectiveness of practices when knowing and distinguishing healthy versions of healthy eating.

This is also the first study that considers self-compassion in relation to orthorexia nervosa. The findings are novel as they indicate that individuals who experience high levels of orthorexic behaviours display low levels of self-compassion. Past research has demonstrated that self-compassion has been related to a number of positive eating behaviours; individuals with higher levels of self-compassion tend to display lower levels of disordered eating [82], and showed more intuitive eating, where they rely on satiety cues and lower dietary restraint [83]. Furthermore, it was also shown that high self-compassion was linked to more mindful eating, lower disordered eating, and lower BMI [56, 63, 84]. Recent studies have shown a clear link between self-compassion and mindful eating [54, 85], however, this was not replicated in the current study as mindful eating was not significantly related to self-compassion in this population. In addition, in the present study, a significant negative relationship was observed between self-compassion and BMI. The results support prior research that identified that high self-compassion is linked to lower BMI [55, 84, 86, 87], but literature on self-compassion and BMI is mixed. There are contradictory findings that shows self-compassion is not related to BMI [56, 63, 88]. As explained by Mantzios and Egan [89] this could be due to self-compassion and self-kindness capturing the eating of unhealthy foods as a method of being kind to oneself. There seems to be a case worth exploring further whether self-compassion is useful for self-regulation of weight and disordered eating, where the potential of looking into aspects of self-kindness for body and mind unlock an element of holistic self-care, as seen in previous literature [85]. Despite the mixed findings, a future study may use a qualitative approach to explore more clearly the utility of self-compassion (and mindful eating) for orthorexia and people who follow a vegan diet.

In addition, a mediation analysis was conducted, where restrictive eating and orthorexia nervosa was explained through self-compassion as a mediator. A significant mediation was observed in the present study, which to date, is an original finding that has not been previously explored, and is consistent with previous literature (e.g., [66]). The present findings indicate that self-compassion interventions could be a useful addition to support individuals who engage in restrictive eating and display orthorexic behaviours. Further studies are needed to expand on the preliminary results of this work.

### Limitations

A clear limitation of this study is the low numbers of males included; this is partially reflective of the general population, where females are more likely than males to become vegans [13]. Gender differences are consistently observed in eating pathology [90, 91] and ON symptomology has been shown to be greater in men than women [92, 93]. Future research on dietary choices including vegan diet and ON in males is warranted as research evidence suggests the role of masculine identity may influence eating behaviours, for example eating a meat-based diet [94] or following a diet specifically aimed at achieving a more muscular body [95].

Motivational factors to consume a vegan diet vary and may include ethical and environmental imperatives or health and weight regulation reasons [96]. Future research should establish motivational factors to offer further knowledge that suggests that associations between following a vegan diet and problematic eating behaviours including ON are linked to health motivations rather than ethical or environmental reasons [81]. While individuals with an eating disorder were discouraged from taking part in the study, we did not directly ask participants to disclose their medical status in this regard and therefore cannot discount the possibility that some participants with a current eating disorder may have been included. Future studies could usefully include this in demographic information.

## Conclusions

The present study has produced some novel findings that add to the research on orthorexia nervosa. This study has demonstrated that self-compassion is negatively associated with orthorexia nervosa, orthorexia is positively associated with restrained eating, and self-compassion mediates the relationship between restrictive eating and orthorexia nervosa. The potential benefits are apparent, as self-compassion could offer an effective tool in treating restrictive eating and orthorexia in those who follow a vegan diet. While self-compassion fits in with predictions made for the present research and past literature in orthorexia, the differential association of BMI to self-compassion and mindful eating propose a potential aspect of weight regulation that has not been identified elsewhere, signifying a unique diverging relation of two mindfulness-based constructs in a vegan population. In other words, when eating mindfully is associated to increased weight, the implications for people who are following a vegan diet may be the inclination to eat inattentively (instead of attentively) and without any physiological correspondence to hunger and satiety (but rather driven by emotions and environmental cues). While there are more questions that derived from the present research than solutions, it offers a steppingstone to explore the nature of engagement with mindful eating in the presence of a vegan diet.

### What is already known on this subject?

Previous studies provided clear evidence that individuals who consume a plant-based diet can display orthorexic eating behaviours. Some literature explored plant-based populations and the association of orthorexia to restrained eating, emphasising the problematic existence of eating behaviours.

### What does this study add?

This study explored orthorexic behaviours by recruiting a vegan population only, assuming a significant association between orthorexia and restrained eating. Findings suggested that high levels of orthorexic behaviours are associated with high levels of restraint and low levels of self-compassion. Furthermore, self-compassion acted as a mediating factor between restrained eating and orthorexia, proposing a potential solution for clinical practice.

## Data Availability

The data sets generated during and/or analysed during the current study are available from the corresponding author on reasonable request.
